# Critical evaluation of the undergraduate curriculum—are we asking the right questions?

**DOI:** 10.1002/ski2.67

**Published:** 2021-09-20

**Authors:** A. Salava

**Affiliations:** ^1^ Department of Dermatology, Allergology and Venereology University of Helsinki and Helsinki University Hospital Helsinki Finland

## Abstract

The curricular content in medical education needs continuous development and therefore must regularly undergo a critical evaluation. Here, the author describes an implemented shift in the teaching substance of an undergraduate dermatology course aimed to focus on relevance and practicability for general practitioners. The changes were based on a comprehensive nationwide database analysis of the spectrum of skin‐related conditions seen in primary care.

1

Dermatology teacher, have you critically evaluated the content of your undergraduate curriculum?

The curricular content of undergraduate medical education is increasingly growing and has a continuous need for development and critical evaluation.^1^ The ongoing COVID‐19 pandemic has turned out to be a notable catalyser of this process.^2^ Particularly in subspecialty undergraduate courses, it has become difficult to handle all the instructional materials during contact teaching and omission of material is inevitable. This arises essential questions for design and implementation of future curricula:

1. What are important disease entities that should constitute the actual core of the teachings?
2. On the other hand, what belongs to material that is more irrelevant and possible to omit on purpose?
3. Is the curricular material grounded on the table of contents of textbooks, or perhaps the individual expertise of the teacher‐specialist, or does it reflect relevant material students will need during their early development as medical doctors?

In recent years, the author has tried to answer the presented questions concerning the undergraduate dermatology courses and voluntary courses on observational skills of the University of Helsinki, Finland. To critically evaluate any undergraduate medical teaching it is vital to explore the spectrum of entities seen in primary care and other non‐specialist settings. Based on nationwide database information, the aim, as a medical specialist, was to acquire a comprehensive picture of the spectrum of skin‐related conditions in primary care and to structure the curricular content accordingly.^3^ The search showed that a limited amount of diagnoses and clinical problems comprise most of the skin‐related conditions in primary care in Finland, namely skin infections, eczematous eruptions and benign skin neoplasms.

These were now shifted to represent the most important material of the curriculum and trained intensively with bedside teaching, problem‐orientated learning and small group teaching.^4^ The idea was to emphasise relevancy in primary care and to highlight what is frequent, and what is not.^5^ On the other hand, the analysis showed that there is a wide spectrum of rarer skin‐related conditions encountered in primary care. These were incorporated in perceptive learning modules and internet‐based learning tools. Resources of contact teaching were however explicitly allocated for the more relevant core materials.

Because medical specialists of university clinics are the main teachers in undergraduate medical education and responsible for conducting practical trainings, there may be substantial discrepancies to the spectrum of conditions seen in primary care and other non‐specialist institutions. The author would like to encourage particularly teachers of smaller subspecialties, such as dermatology, to critically evaluate their curricular content and ask themselves the same questions the author has asked himself.

## FUNDING INFORMATION

No funding resources supported this work.

## CONFLICTS OF INTEREST

The author declares no conflicts of interest.

## AUTHOR CONTRIBUTIONS

**A. Salava:** Conceptualization; Data curation; Formal analysis; Investigation; Methodology; Project administration; Resources; Validation; Visualization; Writing – original draft; Writing – review & editing.

2

## Data Availability

The data that support the findings of this study are openly available: https://doi.org/10.1002/ski2.53.
